# Sex- and Sport-Specific Differences in Jump Strategies: Key Qualities for Jump Performance

**DOI:** 10.3390/sports13090292

**Published:** 2025-09-01

**Authors:** Jing-Hong Lin, Shayna Goldstein, E. Todd Schroeder

**Affiliations:** 1 Olympic Sports Performance, Department of Athletics, University of Southern California, Los Angeles, CA 90089, USA; jinghong@usc.edu; 2 Clinical Exercise Research Center, Division of Biokinesiology and Physical Therapy, School of Dentistry, University of Southern California, Los Angeles, CA 90089, USA; shayna@k2sciences.com

**Keywords:** athlete monitoring, force plate, repeated measures correlation, team sport

## Abstract

This study investigated countermovement jump (CMJ) strategies among NCAA Division 1 athletes and explored key variables associated with jump height. A total of 69 athletes (38 male, 31 female) from basketball and volleyball teams completed three or more CMJ trials on force plates during their regular neuromuscular monitoring. Using repeated-measures correlation analysis, we examined the relationships between various force–time variables and jump height across different sports and sexes. The results demonstrated very strong correlations between concentric peak velocity and jump height across all groups (r > 0.987). In addition, female athletes exhibited higher correlations between force-related parameters (concentric peak force, relative concentric peak force, and relative concentric mean force) and jump height compared to male athletes. Furthermore, no significant differences in force asymmetry were observed between sports or sexes. These findings indicate that concentric peak velocity serves as a key indicator of jump performance while emphasizing the importance of considering the interaction between force, time, and velocity, rather than focusing solely on peak force production. This research provides valuable insights for developing sport-specific training programs and monitoring jump performance in collegiate athletes, highlighting the necessity of individualized assessment and training approaches rather than assuming specific physical qualities are associated with particular populations.

## 1. Introduction

Monitoring countermovement jump (CMJ) performance is a practical method for practitioners to assess an athlete’s neuromuscular status [1,2]. In team sports, such testing is often conducted as part of the regular monitoring process and the results can help practitioners understand fluctuations in physical performance resulting from the stress–recovery balance throughout a season [1,3]. The CMJ has also been shown to be a reliable and valid test for assessing jump height and lower body explosive power in athletes [4,5,6], and the developments of jump height and jump performance are crucial for sports such as volleyball and basketball [7,8,9]. Force plates are considered to be a gold standard to measure a variety of force–time variables of the CMJ, enabling the detailed analysis of jump mechanics [10,11]. Sex differences in CMJ performance often stem from variations in muscle architecture and neuromuscular recruitment patterns [12,13]. For instance, males typically exhibit higher concentric impulse leading to greater velocity throughout the concentric phase [14]. Additionally, sex- and sport-specific differences in jump strategy have been reported [15,16], and the differences in jump strategy may play a crucial role in training program design. In volleyball, the absence of direct physical confrontation requires the indirect optical regulation of the ball’s trajectory and opponent movements, demanding prolonged ground contact times for optimal timing at the jump’s apex during spikes or blocks [15,17,18,19]. In contrast, basketball involves direct opponent confrontation in a constrained environment, which leads to a shorter response time. These differences may manifest in distinct force–time characteristics, such as sustained impulse in volleyball for propulsion or velocity emphasis in basketball for quick recoveries. Previous studies on jump strategies have primarily focused on performance-related force–time variables [20,21]. Meanwhile, the effects of sport demands on limb asymmetry are also an important component of the CMJ [22,23]. Understanding the differences in jump strategy could establish the foundation for coaches and trainers to develop an efficient training program [24]. Defining the dominant variables that underlie jump strategy is essential to explain the variance in jump performance and to prescribe tailored training programs for different sports teams. Therefore, the aim of this study was to investigate whether the sex- and sport-specific differences would result in different jump strategies, specifically to understand if male athletes would show stronger correlations between force-related variables and jump height compared to female athletes, and whether volleyball players would demonstrate different force–time characteristics compared to basketball players due to sport-specific movement demands. The overall goal was to investigate differences in jump strategy with data collected from four teams (men’s and women’s basketball and volleyball) and explore the key qualities for improving jump height.

## 2. Materials and Methods

### 2.1. Experimental Approach

A total of 68 NCAA Division 1 athletes (38 male, 30 female) who had completed three or more CMJ sessions were included in the analysis. Athletes completed a mean of 17 CMJ testing sessions (SD = 6), providing multiple data points per individual for repeated-measures correlation (RMCORR) analysis. The tests were part of the regular neuromuscular monitoring process. Athletes jumped once a week at the beginning of the week. For men’s basketball, when beginning-of-week testing was not feasible due to competition schedule and logistics, athletes jumped before games. A trained sport scientist collected the data. Data were collected using ForceDecks Max (Vald Performance, Newstead, Australia) force plate system at 1000 Hz. The ForceDecks were leveled and zeroed before each testing session. The standardized warmup was instructed by the strength and conditioning specialist and was tailored for the needs of each team. It included jumping, running, and unloaded barbell movements [25,26]. After a standardized warmup, athletes were instructed to step on the ForceDecks and stand still to obtain a baseline bodyweight. They were then instructed to quickly squat to a self-selected depth and then jump as high as they could with their hands on their hips. A self-selected countermovement depth was used to limit protocol intervention and external influence on jump strategy [1,27]. They completed three CMJs each session with 5 s rest between each jump, and the average of three jumps was calculated. Jump testing followed standardized protocols consistent with methods used by Heishman et al. [2], who referenced established reliability for these procedures in collegiate basketball players [28,29]. The variables of interest are outlined in Table 1.

### 2.2. Statistical Analysis

Data were analyzed using Python (version 3.9). The athletes were separated into four groups for analysis based on sex (men and women) and sport type (basketball and volleyball). RMCORR was used to determine the correlation between different force–time variables and jump height (impulse-momentum, cm). Our study used RMCORR to examine the results of CMJ tests throughout the competitive season and preparatory period. Unlike simple regression/correlation, RMCORR does not violate the assumption of independence of observations and is able to examine the scenario that each individual provides more than 1 data point and to accommodate unbalanced designs [30]. Therefore, RMCORR is the ideal method to examine the data collected from regular athlete monitoring processes.

One-way ANOVA was used to assess the difference in asymmetry (percentage in absolute number) during the CMJ tests between sports and sex, aligning with our research questions that did not hypothesize interaction effects between these factors. All available sessions were averaged for each athlete for ANOVA analysis. Correlation coefficients were defined as 0.00–0.10 = negligible, 0.10–0.39 = weak, 0.40–0.69 = moderate, 0.70–0.89 = strong, and 0.90–1.00 = very strong. Statistical significance was set at *p* < 0.05.

## 3. Results

Correlations between various force–time variables and jump height were examined, presented in Table 2 and Table 3. The results showed that there was a very strong correlation between concentric peak velocity and jump height across all groups. Specifically, the correlation coefficient was r = 0.987 for the women (both sports) and r = 0.991 for the men (both sports) while it was r = 0.989 for both the volleyball and basketball teams. The correlations were strong when we consider the average velocity during concentric phase and jump height (men: r = 0.792, women: r = 0.763, volleyball team: r = 0.758, basketball team: r = 0.790).

In contrast, the correlation between concentric peak force and jump height was negligible to moderate (men: r = 0.188, women: r = 0.337, volleyball team: r = 0.438, basketball team: r = 0.089). The correlation between relative concentric peak force and jump height was also weak to moderate (men: r = 0.186, women: r = 0.408, volleyball team: r = 0.423, basketball team: r = 0.136).

The correlation between concentric mean force and jump height showed weak-to-moderate positive correlations (men: r = 0.44, women: r = 0.374, volleyball team: r = 0.513, basketball team: r = 0.378). When we normalized concentric mean force with bodyweight, the correlation with jump height became moderate (men: r = 0.462, women: r = 0.516, volleyball team: r = 0.552, basketball team: r = 0.434).

Meanwhile, the correlation between concentric impulse and jump height was strong to very strong (men: r = 0.881, women: r = 0.867, volleyball team: r = 0. 819, basketball team: r = 0.922).

The correlation between concentric peak power and jump height was strong (: r = 0.755, women: r = 0.698, volleyball team: r = 0.754, basketball team: r = 0.749). The correlations were all increased with four groups when we normalized the peak power with bodyweight (men: r = 0.797, women: r = 0.796, volleyball team: r = 0.833, basketball team: r = 0.777).

When we investigated the power throughout the concentric phase, the correlations were within the same range as peak power. The Mean Concentric Power (men: r = 0.756, women: r = 0.705, volleyball team: r = 0.793, basketball team: r = 0.722) and Relative Mean Concentric Power (men: r = 0.772, women: r = 0.781, volleyball team: r = 0.818, basketball team: r = 0.750) both showed strong correlations with jump height. The correlation between the Flight Time Contraction Time Ratio and jump height was moderate (men: r = 0.507, women: r = 0.572, volleyball team: r = 0.573, basketball team: r = 0.488).

Additionally, one-way ANOVA revealed no significant differences in asymmetry variables between groups, with all comparisons showing *p* > 0.05 for Mean Concentric Force Asymmetry, Peak Concentric Force Asymmetry, Mean Eccentric Force Asymmetry, and Peak Eccentric Force Asymmetry when comparing both sex (female vs male) and sport (basketball vs volleyball) groups (Table 4 and Table 5).

## 4. Discussion

Within all groups, the results showed that the concentric peak velocity has a very strong correlation with jump height; it has the highest vertical velocity prior to take-off. The velocity at take-off was slightly lower than the concentric peak velocity. Take-off was defined as the point where force dropped below 20N and where the velocity would be used to calculate the jump height using the impulse-momentum theorem [5]. Concentric peak velocity was determined by the interaction between force and time. A change in the peak velocity during the concentric phase may be a good indicator of the change in jump height [14]. Tracking the concentric peak velocity could be part of the performance monitoring routine to understand the qualities that underlie the changes in jump height [1]. Furthermore, a training program that considers the movement velocity in the concentric phase would be recommended for a holistic training program [31,32,33].

However, the concentric peak force only moderately correlated for volleyball players (r = 0.439) and was negligible for basketball players (r = 0.089). When compared between sexes, it showed weak correlation for both male and female participants, but female participants had a higher correlation (r = 0.349) compared to male participants (r = 0.188). If we normalize peak concentric force by bodyweight, the correlation is r = 0.424 for volleyball players and r = 0.136 for basketball players. The correlation was r = 0.411 for female and r = 0.186 for male participants.

The results for force variables showed that the ability to generate higher peak force during the concentric phase may not be the most important parameter to consider when the goal is to improve jump height [34]. The force application ability throughout the concentric phase would provide more insight; therefore, jump performance would be better reflected by the relative mean force and concentric impulse [11,35]. Concentric impulse is the product of force and time; it accounts for the changes in force and its relationship with time [5,36]. Moreover, concentric peak power, concentric mean force, relative concentric peak power, and relative concentric mean power are also strongly correlated with jump height. Our results suggest that we should consider the changes in force and its relationship with time and velocity throughout the whole concentric phase [11].

Unlike previous research that found stronger correlations between jump height and relative concentric mean force in males than females [15,37], the investigators suggested that maximizing force variables is a better strategy for optimizing male’s jump performance compared to that of female within the sexes, but this was contradictory to our findings. Our results showed that the concentric peak force, relative concentric peak force, and relative concentric mean force had higher correlations with jump height in our female athletes but also could affect male athletes’ jump height when bodyweight and the entire concentric phase were considered [38]. Research has also shown that force–velocity characteristics for individuals would be affected by different training methods [31,32,33]. Therefore, the correlation may not imply the difference of muscle architecture between sexes. We also need to consider the differences in the training phase; therefore, incorporating RMCORR to analyze the performance throughout a period of time would give us more information than a single test point [30].

When we compare the difference between sports, both peak and mean velocity, impulse, peak and mean power, and peak and mean relative power during the concentric phase had strong-to-very-strong correlations with jump height. However, for force-related parameters, concentric peak force, concentric mean force, relative concentric peak force, and relative concentric mean force were moderately corelated with jump height in volleyball, but only relative concentric mean force was moderately corelated with jump height in basketball, which showed us, again, the importance of the force application throughout the concentric phase [19,20]. We should focus on the interaction between force, time, and velocity [11,39,40,41].

Another finding of this study was that there were no significant differences in both concentric and eccentric force asymmetry between sports and sexes. This result suggested that even volleyball players have more repetitive jumping and less movement diversity; unlike in basketball, which involves both jumping and sprinting [2,15,42], the volleyball players in this study did not develop sports-specific asymmetry compared to basketball players [23]. There are many factors that underlie the development of physical characteristics and testing would provide the most accurate method to understand athletes.

While novel, it is important to acknowledge the limitations inherent in our research design and methodology. The data were collected across the Fall and Spring semesters; therefore, some athletes did not participate in the whole data collection period and had fewer data points. Additionally, some athletes were excluded from our regular countermovement jump tests due to injury. Another limitation was the training program. Each team has different needs, and each strength and conditioning specialist has different approaches for training. The training programs were not the same for each team and had been designed for different situations based on the time of the season and their needs. Also, the cadence and timing of the CMJ tests were different for these teams depending on the competition schedule and logistics, and the CMJ depth was self-selected to limit protocol intervention and external influences on jump strategy, but it may have introduced variability in countermovement depths; therefore, future research with standardized depths could offer additional perspectives on force–time characteristics.

## 5. Conclusions

In conclusion, this study showed that concentric peak velocity is a critical driver of countermovement jump height in NCAA Division 1 basketball and volleyball athletes, with very strong correlations across sex and sport groups. Surprisingly, female athletes had stronger links between force-related variables and jump performance than males, and no notable differences in force asymmetries appeared between sexes or sports. These findings indicate that concentric peak velocity serves as a key indicator of jump performance while emphasizing the importance of considering the interaction between force, time, and velocity rather than focusing solely on peak force production. This research provides valuable insights for developing sport-specific training programs and monitoring jump performance in collegiate athletes, highlighting the necessity of individualized assessment and training approaches rather than assuming that specific physical qualities are associated with particular populations. Above all, it stresses the value of regular testing with tools like force plates and repeated-measures correlations to truly understand athletes’ neuromuscular traits and refine strategies accordingly.

## Figures and Tables

**Table 1:** Description of the countermovement jump variables.

Parameters	Description
Concentric Peak Velocity (m/s)	Highest vertical velocity achieved during the concentric phase
Concentric Mean Velocity (m/s)	Average velocity during the concentric phase
Concentric Peak Force (N)	Highest force achieved during the concentric phase
Concentric Mean Force (N)	Average force during the concentric phase
Relative Concentric Peak Force (N/kg)	Highest force achieved during the concentric phase relative to body mass
Relative Concentric Mean Force (N/kg)	Average force during the concentric phase relative to body mass
Concentric Impulse (N·s)	Area under the NET force–time curve during the concentric phase
Concentric Peak Power (W)	The maximum power value in the concentric phase
Concentric Mean Power (W)	The average power in the concentric phase
Relative Concentric Peak Power (W/kg)	The maximum power value in the concentric phase relative to body mass
Relative Concentric Mean Power (W/kg)	The average power in the concentric phase relative to body mass
Flight Time: Contraction Time	Ratio of flight time to contraction time
Mean Concentric Force ASYM (%)	Asymmetry in the average left and right limb forces in the concentric phase
Peak Concentric Force ASYM (%)	Asymmetry in the peak left and right limb forces in the concentric phase
Mean Eccentric Force ASYM (%)	Asymmetry in the average left and right limb forces in the eccentric phase
Peak Eccentric Force ASYM (%)	Asymmetry in the peak left and right limb forces in the eccentric phase

**Table 2:** Comparison of force–time variables among sexes and sports.

	Female N = 30	Male N = 38	Basketball N = 30	Volleyball N = 38
Concentric Peak Velocity (m/s)	2.53 ± 0.16 r = 0.987 *	2.93 ± 0.17 r = 0.991 *	2.69 ± 0.26 r = 0.989 *	2.81 ± 0.25 r = 0.989 *
Concentric Mean Velocity (m/s)	1.40 ± 0.10 r = 0.763 *	1.62 ± 0.13 r = 0.792 *	1.49 ± 0.16 r = 0.790 *	1.55 ± 0.15 r = 0.758 *
Concentric Peak Force (N)	1700 ± 327 r = 0.337 *	2170 ± 267 r = 0.188 *	2015 ± 425 r = 0.089 *	1921 ± 331 r = 0.438 *
Concentric Mean Force (N)	1370 ± 242 r = 0.374 *	1737 ± 219 r = 0.44 *	1613 ± 335 r = 0.378 *	1544 ± 255 r = 0.513 *
Relative Concentric Peak Force (N/kg)	22.07 ± 1.3 r = 0.408 *	24.51 ± 2.17 r = 0.186 *	23.74 ± 2.70 r = 0.136 *	23.15 ± 1.71 r = 0.423 *
Relative Concentric Mean Force (N/kg)	17.77 ± 0.89 r = 0.516 *	19.61 ± 1.72 r = 0.462 *	19.02 ± 2.08 r = 0.434 *	18.63 ± 1.27 r = 0.552 *
Concentric Impulse (N⋅s)	186 ± 32 r = 0.867 *	250 ± 31 r = 0.881 *	218 ± 46 r = 0.922 *	225 ± 44 r = 0.819 *
Concentric Peak Power (W)	3522 ± 698 r = 0.698 *	5063 ± 771 r = 0.755 *	4401 ± 1196 r = 0.749 *	4370 ± 965 r = 0.754 *
Concentric Mean Power (W)	1800 ± 352 r = 0.705 *	2630 ± 412 r = 0.756 *	2262 ± 624 r = 0.722 *	2266 ± 523 r = 0.793 *
Relative Concentric Peak Power (W/kg)	45.73 ± 4.98 r = 0.796 *	57.11 ± 6.51 r = 0.797 *	51.62 ± 9.71 r = 0.777 *	52.46 ± 6.81 r = 0.833 *
Relative Concentric Mean Power (W/kg)	23.38 ± 2.49 r = 0.781 *	29.72 ± 4 r = 0.772 *	26.62 ± 5.5 r = 0.750 *	27.16 ± 3.91 r = 0.818 *
Flight Time: Contraction Time	0.58 ± 0.06 r = 0.572 *	0.69 ± 0.09 r = 0.507 *	0.63 ± 0.11 r = 0.488 *	0.64 ± 0.08 r = 0.573 *

Mean ± SD values of force–time variables are shown, and r values are the correlations compared to jump height, * *p* < 0.05.

**Table 3:** *p*-value and power of force–time variables among sexes and sports.

	Female N = 30	Male N = 38	Basketball N = 30	Volleyball N = 38
Concentric Peak Velocity (m/s)	*p* = ~0, power = 1	*p* = ~0, power = 1	*p* = ~0, power = 1	*p* = ~0, power = 1
Concentric Mean Velocity (m/s)	*p* = 2.53 × 10^−87^, power = 1	*p* = 5.72 × 10^−133^, power = 1	*p* = 3.62 × 10^−122^, power = 1	*p* = 4.50 × 10^−94^, power = 1
Concentric Peak Force (N)	*p* = 1.72 × 10^−13^, power = 1	*p* = 2.64 × 10^−6^, power = 1	*p* = 0.03, power = 0.56	*p* = 9.65 × 10^−25^, power = 1
Concentric Mean Force (N)	*p* = 1.87 × 10^−16^, power = 1	*p* = 1.97 × 10^−30^, power = 1	*p* = 1.03 × 10^−20^, power = 1	*p* = 1.16 × 10^−34^, power = 1
Relative Concentric Peak Force (N/kg)	*p* = 1.34 × 10^−19^, power = 1	*p* = 3.77 × 10^−6^, power = 1	*p* = 0.0011, power = 0.90	*p* = 6.05 × 10^−23^, power = 1
Relative Concentric Mean Force (N/kg)	*p* = 3.62 × 10^−32^, power = 1	*p* = 1.10 × 10^−33^ power = 1	*p* = 1.88 × 10^−27^, power = 1	*p* = 6.57 × 10^−41^, power = 1
Concentric Impulse (N⋅s)	*p* = 2.99 × 10^−138^, power = 1	*p* = 5.18 × 10^−201^, power = 1	*p* = 8.16 × 10^−236^, power = 1	*p* = 1.14 × 10^−121^, power = 1
Concentric Peak Power (W)	*p* = 2.89 × 10^−67^, power = 1	*p* = 2.80 × 10^−114^, power = 1	*p* = 4.67 × 10^−103^, power = 1	*p* = 1.58 × 10^−92^, power = 1
Concentric Mean Power (W)	*p* = 3.68 × 10^−69^, power = 1	*p* = 8.85 × 10^−115^, power = 1	*p* = 1.27 × 10^−92^, power = 1	*p* = 2.11 × 10^−108^, power = 1
Relative Concentric Peak Power (W/kg)	*p* = 4.86 × 10^−100^, power = = 1	*p* = 3.43 × 10^−136^, power = 1	*p* = 1.13 × 10^−115^, power = 1	*p* = 1.70 × 10^−129^, power = 1
Relative Concentric Mean Power (W/kg)	*p* = 6.05 × 10^−94^, power = 1	*p* = 3.14 × 10^−122^, power = 1	*p* = 1.10 × 10^−103^, power = 1	*p* = 5.52 × 10^−121^, power = 1
Flight Time: Contraction Time	*p* = 1.25 × 10^−40^, power = 1	*p* = 1.95 × 10^−41^, power = 1	*p* = 2.22 × 10^−35^, power = 1	*p* = 9.29 × 10^−45^, power = 1

**Table 4:** Comparison of asymmetry variables among sexes and sports.

	Female N = 30	Male N = 38	Basketball N = 30	Volleyball N = 38
Mean Concentric Force ASYM (%)	5.43 ± 2.88	6.46 ± 4.09	5.71 ± 3.96	6.23 ± 3.36
Peak Concentric Force ASYM (%)	4.92 ± 2.40	6.05 ± 3.42	5.01 ± 2.84	5.98 ± 3.16
Mean Eccentric Force ASYM (%)	8.91 ± 4.62	9.54 ± 3.67	9.29 ± 3.68	9.24 ± 4.45
Peak Eccentric Force ASYM (%)	7.82 ± 4.96	9.66 ± 4.85	8.58 ± 4.87	9.06 ± 5.06

Mean ± SD values of asymmetry variables are shown; asymmetry was calculated as percentage for each group.

**Table 5:** *p* values: comparison of asymmetry variables between groups using one-way ANOVA.

	Female vs. Male	Basketball vs. Volleyball
Mean Concentric Force ASYM (%)	*p* = 0.249	*p* = 0.561
Peak Concentric Force ASYM (%)	*p* = 0.131	*p* = 0.193
Mean Eccentric Force ASYM (%)	*p* = 0.528	*p* = 0.963
Peak Eccentric Force ASYM (%)	*p* = 0.130	*p* = 0.698

## Data Availability

The data is available upon request from the corresponding author due to privacy and ethical restrictions.

## Abbreviations

The following abbreviations have been used in this manuscript:

CMJ	Countermovement Jump
NCAA	National Collegiate Athletic Association
RMCORR	Repeated-Measures Correlation
ANOVA	Analysis of Variance
